# The reproduction rate of severe acute respiratory syndrome coronavirus 2 different variants recently circulated in human: a narrative review

**DOI:** 10.1186/s40001-023-01047-0

**Published:** 2023-02-24

**Authors:** Zahra Karimizadeh, Razieh Dowran, Talat Mokhtari-azad, Nazanin-Zahra Shafiei-Jandaghi

**Affiliations:** 1 Students’ Scientific Research Center, Exceptional Talents Development Center, Tehran University of Medical Sciences, Tehran, Iran; 2 Department of Virology, School of Public Health, Tehran University of Medical Sciences, Tehran, Iran

**Keywords:** SARS-CoV-2, Variants of concerns, R_0_, COVID-19, Transmissibility, Basic reproductive number

## Abstract

SARS-COV-2 is responsible for the current worldwide pandemic, which started on December 2019 in Wuhan, China. On March 2020 World Health Organization announced COVID-19 as the new pandemic. Some SARS-COV-2 variants have increased transmissibility, cause more severe disease (e.g., increased hospitalizations or deaths), are resistant to antibodies produced by the previous infection or vaccination, and there is more difficulty in treatment and diagnosis of them. World Health Organization considered them as SARS-CoV-2 variants of concern. The introductory reproduction rate (*R*_0_) is an epidemiologic index of the transmissibility of the virus, defined as the average number of persons infected by the virus after known contact with an infectious person in a susceptible population. An *R*_0_ > 1 means that the virus is spreading exponentially, and *R*_0_ < 1, means that the outbreak is subsiding. In various studies, the estimated R and VOC growth rates were reported to be greater than the ancestral strains. However, it was also a low level of concordance between the estimated Rt of the same variant in different studies. It is because the R of a variant not only dependent on the biological and intrinsic factors of the virus but also several parameters can affect the R0, including the duration of contagiousness and the likelihood of infection per contact. Evaluation of changes in SARS-CoV-2 has shown that the rate of human-to-human transmission of this virus has increased. Like other viruses with non-human sources which succeeded in surviving in the human population, SARS-CoV-2 has gradually adapted to the human population, and its ability to transmit from human to human has increased. Of course, due to the continuous changes in this virus, it is crucial to survey the rate of transmission of the virus over time.

## Background

### SARS-COV-2

Severe acute respiratory syndrome coronavirus 2 (SARS-COV-2) causes coronavirus disease 2019 (COVID-19). This virus belongs to the *Coronaviridae* family and ß-coronaviruses genus, which are enveloped positive-sense single-stranded RNA viruses. The viral genome includes open reading frames (ORF), spike surface glycoprotein (S), membrane (M), nucleocapsid protein (N), and envelope (E). Ribonucleoproteins are packaged into nucleocapsids, and N protein is necessary for viral replication. The membrane protein is a crucial factor affecting virus assembly, and envelope mediates releasing of the virus from host cells by making cation channels. Spike surface glycoprotein is the main protein involved in viral entry into the host cells. Mutations of the S protein are critical and have significant effects on the virus ability to escape from immune system. Despite several mechanisms for reducing the mutations (e.g., RNA proofreading and capping enzymes, such as 3′-5′ exoribonuclease (ExoN), an endoribonuclease (NendoU), and enzymes responsible for capping), these errors are frequently occurred during replication. These mutations are responsible for different variants of SARS-COV-2 [1].

SARS-COV-2 is responsible for the current worldwide outbreak that started on December 2019 in Wuhan, China. Because of its rapidly spreading pattern into several countries, World Health Organization (WHO) declared COVID-19 as the new pandemic on March 2020 [2].

Clinical presentation of COVID-19 infection may vary from asymptomatic to severe and fatal. Most of the patients are asymptomatic and based on a meta-analysis, it was estimated that about 15–16% of them are asymptomatic. The significant percentage of asymptomatic and mildly symptomatic patients leads to a higher percentage of unacknowledged carriers, which is associated with more transmission and rapid spread [3]. However, some may have complications due to pneumonia, and respiratory and multiple organ failure, especially in patients with risk factors like hypertension, diabetes, higher age, chronic cardiovascular or respiratory diseases, and immunodeficiency disorders [2]. The most common symptoms include fever (in 83% of the patients), cough (in 82% of the patients), and shortness of breath (in 31% of the patients). About 2–10% of the patients may experience gastrointestinal symptoms including vomiting, diarrhea, and abdominal pain [4]. Patients may also experience symptoms, including expectoration (28.2% of the patients), fatigue (35.8% of the patients), and headache or dizziness (12.1% of the patients) [5]. The most commonly reported triggers of the headache were stress, drugs, and the infection itself. The pathophysiology of the headache needs to be clearly understood. But this may be caused by direct viral involvement of trigeminal nerve endings in the nasal or oral cavity [6]. Hyposmia is one of the frequently reported symptoms which was reported by 74.2% cases. It is assumed that hyposmia has a higher incidence, as it was also objectively confirmed in 30.1% of the patients who did not report a dysfunction in chemoreception [7]. Less common symptoms include anorexia, dysgeusia, conjunctivitis, and skin lesions. The principal mechanism of lung consolidation during active infection is pulmonary fibrosis. Fibrosis can also occur in the skin and along with other etiologies like coagulopathies, leads to skin lesions in a very low proportion of patients [5, 8]. Analysis suggests that 27–40% of infected patients are asymptomatic and develop no symptoms of infection, around 90% of cases are uncomplicated with moderate symptoms, and 4–7% of cases are hospitalized [2, 9]. The most common laboratory findings include leukocytopenia, increased C-reactive protein (CRP, an acute phase protein), and lymphocytopenia [5].

Fatality indexes, including the infection fatality ratio (IFR) and case fatality rate (CFR), are measured for COVID-19. IFR is defined as the probability of death in infected cases and is used to determine the severity of a pandemic. CFR is defined as the proportion of people who die from a specified disease among all individuals diagnosed with the disease over a certain period. In COVID-19, IFR is much lower than CFR because of the high rate of asymptomatic infections which are not recognized. There is no agreement on the amount of IFR for COVID-19, but the best models estimate this index to be 0.5–1. While the overall CFR value for COVID-19 is 2.3%, 13.8%, 3.8%, 8.2%, and 1.6% in China, Italy, Germany, Spain, and Südkorea, respectively [2].

Some SARS-COV-2 variants are of great importance due to their potential for increased transmissibility, more severe disease (e.g., increased hospitalizations or deaths), reduced effectiveness of antibodies produced by the previous infection or vaccination, lower efficacy of treatments or vaccines, and diagnostic detection failures. WHO declared these variants as SARS-CoV-2 variants of concern (VOC) [10]. Among these properties, transmissibility is one of the prominent characteristics of SARS-CoV-2, which is increasing by passing time. To compare the transmissibility of SARS-CoV-2 circulated variants by passing time, this study aimed to gather and review the reproduction rate of different SARS-CoV-2 variants spreading in humans during the recent two years.

### Epidemiologic indices used to describe SARS-COV-2

The introductory reproduction rate (*R*_0_) is an epidemiologic index describing the transmissibility of a virus, defined as the average number of new infections generated by an infectious person in a susceptible population. The *R*_0_ refers to the initial reproduction number of the virus at the beginning of the pandemic. In estimating *R*_0_, it is considered that there are no external controls against the virus spread. External controls may include lockdown rules in cities, isolation of confirmed infected cases, vaccination, and traffic control which decreases virus spread.

An *R*_0_ > 1 means that the virus is spreading exponentially, and for *R*_0_ < 1, the outbreak is subsiding [11].

It should be noted that the reproduction number at any given time during an epidemic is called Rt or Re (effective reproduction number). It varies as immunization in the population increases following vaccination, infection, and when people die [12]. Reproduction number varies due to biological factors (e.g., viral load) and social factors such as geographic regions and inter-individual variances, which affect the probability of transmission from a single case (e.g., number of contacts). The inter-individual variance can be explained by overdispersion parameter k (kappa). Kappa is in negative correlation with a variance of inter-individual transmission rates. Another critical parameter affecting *R*_0_ is the serial interval (SI), defined as the time between successive cases in a chain of transmission [13].

All these factors have affected SARS-CoV-2 by passing the time, so different variants of this virus have shown different reproduction and transmissibility in the human population, which might provide an opportunity for virus adaptation to the new host. This narrative review looked over SARS-CoV-2 reproduction number variations since the onset of the pandemic.

## Methods

Herein, the search was performed in the PubMed database using the following terms “variants of concern & basic reproduction rate”, “variants of concern & effective reproduction rate”, “variants of concern & growth rate”, and “variants of concern & transmissibility”. All papers published in English since the emergence of SARS-COV-2 until July 15th, 2022, were included in this narrative review. The information of all studies is summarized in Tables 1, 2, 3, 4, 5, 6.

**Table 1:** Summary of studies on the reproduction number of the Wuhan variant

Study	Location	Study date	Method	Estimated R0	Other findings
Ke et al.	United States and eight European countries	March 2020	SEIR type	Between 3.5 and 6.4, with the highest amounts of 5.9 (CI 4.7–7.5) and 6.4 (5.2–8.0) for the US and Spain, respectively	Growth rate: between 0.19–0.29/day doubling time: between 2.4 and 3.7 days
Liu et al.	Based on 12 studies located in China	January 2020 to February 2020	PubMed, bioRxiv and Google Scholar were accessed	R0 was estimated to be between 1.4 and 6.49 (mean = 3.28, median = 2.79, and interquartile range (IQR) = 1.16)	
Zhao et al.	China	January 2020	A method based on the statistical exponential model, adopting SI from SARS and MERS	Between 2.24 (95% CI 1.96–2.55) to 5.71 (95% CI 4.24–7.54)	
Islam et al.	Bangladesh	March 2021 to July 2021	Four different methods based on the reported confirmed cases by the government. Including SB, TD, ML, and EG	EG model: 1.173 (95% CI 1.172–1.174) ML model: 1.09 (95% CI 1.08–1.10) SB model: 1.24 (95% CI 1.18–1.29) TD model: 1.07 (95% CI 1.02–1.13)	CFR: 1.32% overall recovery rate: 56.85%

**Table 2:** Summary of studies on the reproduction number of the alpha variant

Study	Location	Study date	Method	Estimated R0	Other findings
Curran et al.	UK	August, 2020 to December, 2020	Rapid scoping review approach		Transmissibility: 52% (95% CI 46, 58) higher than the other variants Growth rate: 71% (95% CI 65–75%) higher than the other variants
Grabowski et al.	UK	October, 2020 to December, 2020	Based on GISAID data available and Genome Sequence Analysis		Replicative advantage of B.1.1.7 over 20A.EU1: 1.83–2.18 (95% CI 1.71–2.40) Replicative advantage of B.1.1.7 over other strains: 2.03–2.47 (95% CI1.89–2.77)
Hunter et al.	UK	October, 2020 to December, 2020	R for preceding days was obtained by summing all reports of new COVID infections for each day and the previous six days. This was then compared with the sum of new cases over the previous 7-day period		Rt value for B.1.1.7: 1.3, 1.3 and 1.1 Rt value for other strains: 0.92, 0.91 and 0.97 In England, London and South East regions
Davies et al.	UK	March, 2020 to December, 2020	Using a time-varying multinomial spline model		Increased growth rate of + 0.104 day^–1^ [95% CI 0.100 to 0.108] relative to the previous variant 77% (95% CI 73 to 81%) higher R value
Washington et al.	US	December 2020 to January 2021	SARS-CoV-2 sequencing and Phylogenetic analyses		35–46% increased transmissibility
Sheikhi et al.	Iran	April, 2021 to April, 2021	EGR, ML, SB, and SIR models	R0: 2.26–11.38 using statistical models 12.13 by time-dependent SIR model (in the time interval with exponential growth)

**Table 3:** Summary of studies on the reproduction number of the beta variant

Study	Location	Study date	Method	Estimated R0	Other findings
Grabowski et al.	South Africa	October, 2020 to December, 2020	Based on GISAID data available and *Genome Sequence Analysis*		Rt: 1.55 (95% CI 1.43–1.69) The relative advantage over other South Africa strains: 1.58 (95% CI 1.45–1.72)

**Table 4:** Summary of studies on the reproduction number of the gamma variant

Study	Location	Study date	Method	Estimated R0	Other findings
Naveca et al.	Amazonas, Brazil	November, 2020 to January, 2021	Using real-time RT-PCR Ct value as a proxy of viral load		Ct of P.1-positive patients = 19.8 Ct of P.1 negative patients = 23.8
Faria et al.	Amazonas, Brazil	November, 2020 to December, 2020	Dynamic modeling which integrates genomic information and mortality data		Ct lowered by 1.43 (using E genes) and 1.91 (using N genes)

**Table 5:** Summary of studies on the reproduction number of the delta variant

Study	Location	Study date	Method	Estimated R0	Other findings
Li et al.	Guangdong, China	May, 2021 to June, 2021	Calculating viral loads in the oropharyngeal swabs of quarantined individuals by days between exposure and the time of RT-PCR test		Viral loads: 1260-fold higher in delta variant infected patients in comparison to those of the wild-type strain
Liu et al.			Review study	Mean R value of delta variant = 5.08 (between 3.2 and 8) Mean R0 of ancestral variant = 2.79	
Tian et al.			Review study	R value of delta variant = 4.04–5.0 R0 of wild-type variants = 2.2–3.77	GT of the delta variant = 2.9 GT of the wild-type variants = 5.7

**Table 6:** Summary of studies on the reproduction number of the omicron variant

Study	Location	Study date	Method	Estimated R0	Other findings
Khan et al.	South Africa	November 2021 to January 2022	Using a mathematical model using the Omicron feature and the accurate data of infected cases	R value = 2.1107	
Nishiura et al.	Gauteng province, South Africa	end of November 2021	Using the data from GISAID, the effective reproduction number of the Omicron variant (*R*_t_) was calculated by assuming that the *R*_t_ of omicron is given by multiplying a constant factor *k* by that of the Delta variant		*R*_t_ of the omicron variant: 4.2 times (95% CI 2.1, 9.1) higher than the R_t_ of the delta variant
Jalali et al.	Norway	December 2021 to January 2022	Use contact tracing data collected by Norwegian municipalities to estimate and compare household		SAR for Omicron variant: 51% (95% CI 48–54) SAR for delta variant: 36% (95% CI 33–40) RR = 1.41 (95% CI 1.27–1.56) VE for Omicron variant = 45% (CI 95 26–57) VE for delta variant = 65% (CI 95 42–80)
	South Africa	Up to 25 April 2022	The multinomial logistic regression model		Growth advantages for BA.4 over BA.2 = 0.08 (95% CI 0.07–0.09) Growth advantages for BA.5 over BA.2 = 0.12 (95% CI 0.09–0.15)

SAR, secondary attack rate; RR, relative ratio; VE, vaccine efficacy

### Wuhan variant

Wuhan variant was the first variant of SARS-COV-2 detected in December 2019 in Wuhan, China (Table 1). A study tried to develop and fit a mathematical model based on the morbidity and mortality data of this variant in the United States and eight European countries during the early periods of COVID-19 epidemics to estimate the growth rate of the epidemics and used this estimated growth rate to compute the *R*_0_ of each country. They constructed a Susceptible–Exposed–Infectious–Removed (SEIR) type model using ordinary differential equations, which used the estimated growth rate, the duration of latency (the period between being infected and becoming infectious), and infection as inputs. The latency duration and the infection period was assumed to be 3–4 days and 6–8 days, respectively. The estimated growth rates of nine countries ranged between 0.19 and 0.29/day, which can be interpreted as doubling times between 2.4 and 3.7 days. The highest growth rates were estimated for Spain and US, which were 0.29/day and 0.28/day, respectively. In accordance with these data, the estimated *R*_0_ of nine countries ranged between 3.5 and 6.4 with the highest amounts of 5.9 (Confidence interval CI 4.7–7.5) and 6.4, (5.2–8.0) for the US and Spain, respectively [8].

In another review study by Liu et al. based on 12 studies between 1 January 2020 and 7 February 2020, the *R*_0_ was estimated to be between 1.4 and 6.49 (mean = 3.28, median = 2.79, and interquartile range (IQR) = 1.16]. The low level of concordance between the studies might be due to the difference in considered variables, methods of modeling, and estimation procedures [11].

Zhao et al. used a method based on the statistical exponential model adopting SI from severe acute respiratory syndrome SARS (mean = 8.4 days, SD = 3.8 days) and Middle East Respiratory Syndrome MERS (mean = 7.6 days, SD = 3.4 days). This model used the data released by the Wuhan Municipal Health Commission, China, and the National Health Commission of China from January 10 to January 24, 2020. The *R*_0_ was estimated to be between 2.24 (95% CI 1.96–2.55) to 5.71 (95% CI 4.24–7.54) [14].

In a study based on the incidence data of 145 days (March 8 to July 31, 2020) in Bangladesh, the case fatality rate was estimated to be 1.32% of cases with a confirmed diagnosis, and the total recovery rate was calculated at 56.85%. They calculated the *R*_0_ using four different methods based on the confirmed cases reported by the government. The methods include Sequential Bayesian (SB), time-dependent (TD), Maximum likelihood (ML), and exponential growth (EG). Using the EG, ML, SB, and TD methods based on the data collected between March 8 to July 31, 2020, the *R*_0_ was estimated to be 1.173 (95% CI 1.172–1.174), 1.09 (95% CI 1.08–1.10), 1.24 (95% CI 1.18–1.29), and 1.07 (95% CI 1.02–1.13), respectively. In this study, the *R*_0_ had variation by the progression of time and had the highest value in and around 20–25th days. The highest *R*_0_ was calculated during the fourth week (29 March–4 April) (R_0_ = 4.86), and the lowest R_0_ was in the 20th week (19–25 July) (R_0_ = 1.09) [15].

### Alpha variant

Variant B.1.1.7 first emerged in the UK in late 2020, and from that time till March 9th, 2021, it has spread in 111 countries, and within four weeks, it became the dominant variant by January 21st and reached 92% after two more weeks [16] (Table 2). This variant is characterized by 17 mutations, such as several mutations of the spike protein that mediates virus attachment and entry into human epithelial cells. There is supporting evidence that mutation N501Y enhances binding affinity to human angiotensin-converting enzyme 2 (ACE2); it was around 52% (95% CI 46–58) more transmissible than the 501N variant [17]. Besides, P681H mutation can affect both infectivity and transmissibility [18].

Based on the data from the UK obtained between August 1st and December 31st, 2020, the B.1.1.7 was estimated to be 52% (95% CI 46, 58) more transmissible than the Wuhan variant. The growth rate of this variant is estimated to be 71% (95% CI 65–75%), higher than the predecessor lineage [16].

In another study in the UK based on the data between October 19th to December 20th, 2020, the replicative advantage of variant B.1.1.7 over 20A.EU1 (the previous dominant variant) was estimated to be between 1.83 and 2.18 (95% CI 1.71–2.40), and the estimated replicative advantage for B.1.1.7 concerning the other strains was between 2.03 and 2.47 (95% CI 1.89–2.77). This study, was also demonstrated that the substitution of the spike protein L18F was a mutation of significance because this mutation made this variant resistant to the neutralizing antibodies, which could increase its transmissibility and speed its spread. The fraction L18F substitution in the expanding 20A. EU1 strain was increasing very slowly, and from a fraction of 35% (1332 out of 3799) in September, it reached a value of 52% (8917 out of 17,470) in November 2020 [19].

Hunter et al. reported that starting from the isolation rules in November to the first days of December, despite the reduction in all other strains, B.1.1.7 increased with an R_t_ value of 1.3, 1.3, and 1.1 in the east of England, London, and South East regions, respectively. The R_t_ value for all other strains in these three regions was estimated to be 0.92, 0.91, and 0.97, respectively [20].

In the United States, based on the data from December 2020 to January 2021, B.1.1.7 was estimated to have 35–46% more transmissibility in the US, with rates of 29–37%, and 38–49% in California and Florida, respectively. In this study, it was shown that the majority of the B.1.1.7 genomes from Florida contained K1191N and Q493K mutations. There was evidence that these mutations might contribute to the potential of immune escape [21].

In a study in the UK using a time-varying multinomial spline model based on the data from March to December 2020, VOC B.1.1.7 lineage has estimated to have an increased growth rate of + 0.104 day^–1^ [95% CI 0.100 to 0.108] relative to the B.1.177 variant, which was previously dominant. This increase in growth rate yielded a 77% (95% CI 73–81%) higher R-value. This is in concordance with the data from other countries; the estimated *R* for B.1.1.7 relative to other lineages was 55% (95% CI 45–66%) higher in Denmark, 74% (95% CI 66–82%) higher in Switzerland, and 59% (95% CI 56–63%) higher in the United States. It was hypothesized that the higher transmissibility might be due to lower cycle threshold (Ct) values in real-time PCR tests (which means higher viral loads), the longer period of viral shedding, and the possibility of a higher potential to escape the immune system. Ct is defined as the number of cycles necessary for the fluorescent signal and the presence of the virus to be detected [22].

In a study in Iran, three statistical models and one mathematical model were used to calculate the R value of alpha and delta variants. These models included the Exponential Growth Rate (EGR), Maximum Likelihood (ML), Sequential Bayesian (SB), and time-dependent SIR model. Based on the data from March 10, 2021, until June 10, 2021, the total R-value of the alpha variant was estimated to be 0.9999 (95% CI 0.9994–1), 1.046 (95% CI 1.044–1.049), 1.06 (95% CI 1.03–1.08), and 2.79 (95% CI 2.77–2.81) using EGR, ML, SB, and SIR models, respectively. It should be noted that during the period of exponential growth of alpha variant in Iran (3 to April 9, 2021), the R index of this variant was estimated to be 2.26 (95% CI 2.04–2.49), 2.64 (95% CI 2.58–2.7), 11.38 (95% CI 11.28–11.48), and 12.13 (95% CI 12.12–12.14) using SB, EGR, ML, and SIR models, respectively [23].

### Beta variant

The second variant of concern B.1.351 contained a variety of mutations, such as a mutation in spike protein N501Y that was associated with higher transmissibility. This variant is also known as the 501Y.V2 strain. This variant was first detected in South Africa in early August 2020 and began to spread rapidly. As of January 10, 2021, eight countries had reported cases with the B.1.351 variant and as of March 9th, 2021, B.1.351 has been reported in 58 countries across all six WHO regions. In a study based on the data from South Africa in weeks 43–50 of 2020, the R_t_ value for 501Y.V2 strain was 1.55 (95% CI 1.43–1.69), which was 55% higher than the equivalent value of the wild-type variant (Wuhan). The relative advantage of this variant was estimated to be at a weekly rate of 1.58 (95% CI 1.45–1.72) over the other South African strains [19].

According to CDC reports, the detection of B.1.351 happened at the same time as a rapid rise in the number of cases in Zambia, suggesting an epidemiologic linkage between COVID-19 outbreaks in Zambia and South Africa. CDC also stated that B.1.351 might be associated with higher viral loads. However, they did not report more specifics of transmissibility [24] (Table 3).

### Gamma variant

P.1 variant (evolved from a local B.1.1.28, formerly called B.1.1.28.1; also known as N501Y.V3) was detected for the first time in Manaus, Brazil, on December 4th, 2020, with a high-speed increasing prevalence up to January 2021. Based on WHO reports, as of March 9th, 2021, this variant was detected in 32 countries and all six WHO regions. P.1 contained 21 lineage-defining mutations, containing ten mutations in the Spike proteins (L18F, T20N, P26S, D138Y, R190S, K417T, E484K, N501Y, H655Y, and T1027I) that were associated with higher affinity to bind to the human ACE2 and this greatly affected the transmissibility of this variant. P.1 also harbors Non-Structural Protein 6 (NSP6) deletion, and based on the data of positive samples of SARS-CoV-2 sequenced from November 1st, 2020, to January 31st, 2021, none of them included this mutation before December 16th, indicating that P.1 variant was rarely detected in Amazonas up to mid-December 2020. Based on the data from both genomic sequencing and real-time PCR testing, the prevalence of the P.1 variant increased from 0% (*n* = 0/88) in November 2020 to 4% (*n* = 2/54), 45% (*n* = 104/232), and 73% (*n* = 119/162) in December 1st to 16th, December 17th to 31st 2020, and in January 1st to 15th, 2021, respectively. This study used the real-time RT-PCR Ct value as a proxy of viral load. Ct had a lower value in P.1-positive patients (Ct = 19.8) compared to P.1 negative patients (Ct = 23.8) at a similar time. This data was suggestive of a tenfold greater viral loads in P.1-positive patients, exhibiting that P.1-positive patients were more infectious in comparison to those with non-P.1 viruses [25].

In a study based on the sequencing of SARS-CoV-2 genomes from patients in Amazonas, Manaus, between November and December 2020 using dynamic modeling which integrated genomic information and mortality data, the P.1 variant was estimated to have 1.4 to 2.2 times higher transmissibility and was 25–62% more likely to escape the protecting immunity of former infections. This variant had more affinity to bind to the human ACE2 receptor. Previous infection by non-P.1 variants, provided lower protection against P.1 than other variants; this protection was 54–79% of the protection against non-P.1 variant. In this study, infection with P.1 variant was associated with lower Ct values (as a parameter in reverse relation with viral loads) compared to non-P.1 variant. For both the E gene and the N gene, Ct values of P.1 variant lowered by 1.43 [0.17 to 2.60, 95% CI] and 1.91 (0.49 to 3.23) cycles, respectively [26] (Table 4).

### Delta variant

The Delta (Pango Lineage B.1.617.2) variant emerged in India in October 2020 and spread through at least 185 countries. From 20 to 27 May 2021, the confirmed Delta variant cases increased from 3424 to 6959 in the UK. Twenty-three mutations define this variant compared to the first identified strains. Twelve mutations in spike proteins were imperative in the attachment of the virus to the host cells. The most notable mutations of B.1.617.2 included T19R, L452R, T478K, D614G, P681R, and D960N, with deletions at positions 157 and 158. L452R and P681R spike protein mutations were the most important mutations of the spike protein, which enhanced attachment to the ACE2 [10].

In a study by Li et al., performed between 21 May and 18 June 2021 in Guangdong, China, viral loads of SARS-COV-2 infected patients were calculated by days between exposure and the time of RT-PCR test using a generalized additive model. Viral loads of patients infected with the delta variant were reported to be 1260-fold greater in comparison to those who were infected with the wild-type strain (*n* = 62, Ct = 24.00 for the ORF1ab gene, IQR 19.00–29.00 for B.1.617.2 vs. *n* = 63, Ct = 34.31 for ORF1ab gene, IQR 31.00–36.00 for wild-type strains). The rate of delta variant infected patients with viral loads in oropharyngeal swabs higher than 6 × 10^5^ copies/mL was 80.65% which was remarkably higher than 19.05% in wild-type strains infected patients [22]. Furthermore, the mean viral shedding time was higher in patients infected with the delta variant compared to wild-types (13–15 days vs. 7–9 days)[10].

In an analysis based on five studies from January 2020 to 30 July 2021, the R-value of the delta variant was estimated to range from 3.2 to 8. The mean was estimated to be 5.08, which was higher than the R_0_ of the ancestral strain, with a mean of 2.79 estimated in another review study by Liu et al. The estimated R values in these five studies were reported to be 3.2 (95% CI 2.0–4.8; Guangdong, China), 4.04–5 (Guangdong, China), 5–8 (England), 5.2 (UK), and 6 (China) [27]. In a review study, the incubation period of the delta variant was shown to be 2–3 days, and this time was shorter in comparison to the wild-type strains (3–7 days). In this study, the R-value of the delta variant was estimated to range between 4.04 and 5.0, which was greater than the R0 of the wild-type variants (range from 2.2 to 3.77) [10] (Table 5).

### Omicron variant

Omicron (Pango Lineage B.1.1.529) is the new variant of COVID-19, first observed in South Africa in November 2021 and spread to many European countries. Omicron has caused less severe disease than other variants, including the delta variant, and transmits more efficiently than the other variants [28]. This variant has several mutations with 37 nonsynonymous changes in the spike protein, associated with the enhanced potential to escape the immune system and increased transmissibility. Several mutations in the genome of the omicron variant, including K417N, E484K, N501Y, D614G, and T478K, are shared by other SARS-COV-2 variants. N501Y mutation, which is the substitution of an asparagine residue for tyrosine, has already been detected in the alpha, beta, and gamma variants and is associated with a higher binding affinity to human ACE2 [29].

In a study based on the data from South Africa, between November 1st, 2021, to January 23rd, 2022, the omicron feature was used to develop the model and use the accurate data of the infected cases to estimate the R-value. Rt of the omicron variant was calculated to be 2.1107. Some important factors that could affect the R-value in this model and reduce infection in the population were washing hands, social distancing, and wearing masks [28].

In another study based on the data in Gauteng province, South Africa, using the data as of the end of November 2021, the R_t_ of the omicron variant was calculated by multiplying a constant factor k by the R_t_ of the delta variant. In this study, the R_t_ of omicron was estimated to be 4.2 times (95% CI 2.1, 9.1) higher than the effective reproductive number of the delta variant. The R_t_ of the other variants was 1.3 times (95% CI 0.7, 2.0) greater than that of the delta variant. This higher transmissibility could be explained by the reduced efficacy of current vaccines in neutralizing the omicron variant [30].

Based on the data from the Norwegian COVID-19 pandemic preparedness register, Beredt C19, between December 2021 and January 2022, the omicron variant had a greater secondary attack rate (SAR) compared the delta variant. SAR is defined as the likelihood that an infection occurs among susceptible members of a household or other close-contact environments within enough incubation time after known contact with an infectious person. This value was estimated to be 51% (CI_95_ 48–54) for omicron and 36% (CI_95_ 33–40) for the delta variant, with a relative risk (RR) of 1.41 (95% CI 1.27–1.56). Although the SAR for both omicron and delta variants in booster-vaccinated primary cases was lower compared to booster-unvaccinated primary cases, booster-vaccination had a limited effect in preventing omicron transmission. SAR for omicron in boosted primary cases was estimated to be 46% vs. 11% for the delta variant (RR 4.34; 95% CI 1.52–25.16) [31].

The omicron variant contained three dominant lineages in the first wave in South Africa, including BA.1, BA.2, and BA.3. But in a study by Tegally et al., they identified two new lineages, known as BA.4 and BA.5, which were estimated to be originated mid-December 2021 and early January 2022, respectively. These lineages are characterized by mutations such as 69-70del, L452R, F486V, and the wild-type amino acid at Q493 in spike proteins. In this study, using a multinomial logistic regression model based on the data up to 25 April 2022, growth advantages for BA.4 and BA.5 over BA.2 in South Africa were estimated to be 0.08 (95% CI 0.07–0.09) and 0.12 (95% CI 0.09–0.15) per day, respectively. Mutations of spike amino acids 452, 486, and 493 can influence ACE2 and antibody binding of these variants and lead to increased infectivity [32] (Table 6).

## Discussion

SARS-COV-2 is an RNA virus responsible for the current worldwide pandemic, which started in December 2019 in Wuhan, China. SARS-COV-2 spread over several countries very rapidly. In March 2020, WHO proclaimed COVID-19 as the new pandemic. One of the most discussed features of COVID-19, is its transmission rate in the human population. The reproduction rate is an epidemiologic index to describe the transmissibility of a virus and the spread of the disease. The R0 shows the primary reproduction number of the virus at the beginning of the pandemic or epidemic when there is no control measure. At the same time, Rt refers to the reproduction number at any given time during an epidemic [2].

R0 is estimated using the data of infection in a wholly susceptible population. If *R*0 is below 1, the disease subsides, whereas if *R*0 is greater than 1, the disease persists in that population. R0 is used to calculate the classical herd immunity threshold and the vaccine coverage, which is necessary to achieve herd immunity. R0 varies due to biological factors, social factors, and inter-individual variance of the probability of transmission from a single case [11].

Wuhan strain was the initiating strain in China which was first detected in December 2019 in Wuhan, China [8]. The genome of SARS-COV-2 has undergone various mutations, which led to the emergence of variants of concerns (VOCs) that are highly transmissible and cause more severe disease and even death. These variants may resist to antibodies produced during the previous infection or vaccination. The emergence of VOCs also has led to lower efficacy of treatments or vaccines and diagnostic detection failures. Some of the essential mutations occurred in the genes encoding the spike proteins. These proteins are fundamental modulators of virus attachment, especially to the ACE2, the main receptor for SARS-COV-2 in human cells. This higher affinity to ACE2 may affect VOCs’ transmissibility [11, 15].

In various studies, the estimated R and VOC growth rate were reported to be higher than the ancestral strains. However, it was also a low level of concordance between the estimated Rt of the same variant in different studies. It is because that the R of a variant does not only become dependent on the biological and intrinsic factors of the virus. Several parameters can affect the R0, including the contagiousness duration and the likelihood of infection per contact. Rt is also constantly modified during the epidemic by social factors such as contact rate and control measures like using face masks and lockdowns [13, 27].

## Conclusion

The reproduction numbers of SARS-CoV-2 variants were determined not only by the intrinsic factors of the virus, but also by some social factors and different methods of its calculation. So, the calculated values of R were different in different studies. Since the introduction of SARS-CoV-2 into the human population, the rate of human-to-human transmission of this virus has increased. It seemed that, like other viruses which entered the human population from a non-human population and succeeded to survive in this population, SARS-CoV-2 has gradually adapted to the human population and its ability to transmit from human to human has increased. Of course, due to the continuous changes in this virus, it is crucial to survey the rate of transmission of the virus over time.

## Data Availability

All data generated or analyzed during this study are included in this published article.

## Abbreviations

SARS-CoV-2: Severe acute respiratory syndrome coronavirus 2
COVID-19: Coronavirus disease
WHO: World Health Organization
IFR: Infection fatality ratio
CFR: Case fatality rate
VOC: Variants of concerns
R0: The basic reproduction rate
SI: Serial interval
IQR: Interquartile range
Ct: Cycle threshold
SAR: Secondary attack rate
